# Opposite sex housing enhances reproductive function and induces transcriptional changes in the preoptic area of GnRH-deficient mice

**DOI:** 10.3389/fendo.2025.1571740

**Published:** 2025-04-15

**Authors:** Tyler N. Akonom, Mary A. Allen, Pei-San Tsai

**Affiliations:** ^1^ Department of Integrative Physiology, University of Colorado, Boulder, CO, United States; ^2^ Biofrontiers Institute, University of Colorado, Boulder, CO, United States

**Keywords:** gonadotropin-releasing hormone neurons, fibroblast growth factor receptor, preoptic area, hypothalamic-pituitary-gonadal axis, sexual interactions, reproduction, RNA-Seq - RNA sequencing, GSEA (gene set enrichment analysis)

## Abstract

Sexual interactions have previously been shown to improve reproductive health through unknown mechanisms. In this study, we used RNA-Seq to examine sex-induced gene expression changes in the preoptic area (POA), a critical reproductive brain region. Using a mouse model defective in fibroblast growth factor signaling (dnFGFR mouse), previously shown to disrupt the gonadotropin-releasing hormone (GnRH) system, we examined the impact of opposite sex (OS) housing on gene expression in the POA of a reproductively compromised animal. Bulk RNA-Seq followed by gene set enrichment analysis (GSEA) were used to analyze changes in gene expression and biological processes in control and dnFGFR mice after 300 days of cohabitation with a same sex or OS partner. OS housing of dnFGFR mice, but not control mice, significantly improved reproductive anatomy and gonadotropins in dnFGFR mice. These changes occurred concomitantly with novel biological processes related to estradiol metabolism and neuron excitation. Our results suggest a new role of neuron- or astrocyte-derived estradiol in the plasticity of the GnRH neuron population and offer a promising new direction for the treatment of reproductive disorders stemming from GnRH deficiency.

## Introduction

Sexual interactions have been documented to initiate a myriad of molecular and cellular changes in multiple brain regions (1). These changes include elevated excitatory postsynaptic input, enhanced neurogenesis, gene expression alteration, and dendritic arborization maturation, most of which are considered beneficial for neural network function (1). As such, sexual interactions are widely considered a form of enrichment capable of promoting neural plasticity in adult animals.

An important brain region responsive to sexual enrichment is the preoptic area (POA). In rodents, the medial (m)POA is sexually dimorphic and critical for sexual behaviors (2, 3). It receives sensory input from, and sends efferent projections to, regions controlling precopulatory and copulatory behaviors and the mesolimbic reward circuitry (2, 3). The POA also houses the largest number of neurons producing a critical reproductive neurohormone, gonadotropin-releasing hormone (GnRH). GnRH is a critical activator of the hypothalamic-pituitary-gonadal (HPG) axis. Previous studies have shown that environmental cues with a strong sexual context could activate the HPG axis to acutely release gonadotropins and gonadal steroids in rodents and humans (4–9). Interestingly, rodent studies also showed continuous sexual interactions with opposite sex (OS) cage mates significantly prolonged reproductive lifespan in aging animals (10, 11). These data suggest that stimuli generated from sexual interactions ultimately converged upon the GnRH system in the POA to promote both acute stimulation and chronic maintenance of GnRH neurons. This notion was supported by our previous study demonstrating that OS interactions reversed the decline of detectable GnRH neurons in a mouse model of postnatal GnRH deficiency (12, 13). This mouse model, named dnFGFR, expressed a dominant-negative fibroblast growth factor receptor (dnFGFR) specifically in GnRH neurons, resulting in the functional knockdown of fibroblast growth factor (FGF) signaling in GnRH neurons and an age-dependent decline of detectable GnRH-immunoreactive (ir) neurons after birth (12, 14). Long-term cohabitation of male dnFGFR mice with an OS cage mate completely reversed the decline of detectable GnRH-ir neurons in dnFGFR males and restored their GnRH system to the level of control males (12).

The mechanism underlying OS housing-mediated reversal of the GnRH system in dnFGFR mice is currently unclear. OS interactions could generate multiple olfactory, tactile, visual, and auditory cues. The collective sensory input from OS interactions may subsequently enhance the plasticity of the POA by changing its transcriptional landscape, resulting in the upregulation of pathways neurotrophic to GnRH neurons, and downregulation of pathways detrimental to GnRH neurons. Since GnRH neurons receive very few direct synaptic inputs from other neurons (15), we hypothesized that the restoration of the GnRH system in OS-housed animals was the result of neurotrophic or neurochemical changes in the local POA environment instead of GnRH neurons themselves.

The objective of the current study was to use bulk high-throughput RNA sequencing (RNA-Seq) to analyze genes and biological processes altered by OS housing in the POA of dnFGFR and control male mice. A cohabitation time of 300-days with an OS partner, previously shown to be sufficient for the HPG axis improvement in male dnFGFR mice, was utilized (12). We showed that OS housing enhanced multiple parameters of the HPG axis in dnFGFR but not control male mice, and these changes were accompanied by 22 upregulated and 50 downregulated genes, as well as biological processes associated with cytoplasmic translation, steroid metabolism, and synaptic transmission identified by gene set enrichment analysis (GSEA). These data collectively suggest that sexual enrichment of dnFGFR males induces postnatal plasticity in a key region of the adult brain controlling reproduction. Importantly, molecular mechanisms involved in this plasticity may be exploited to improve the reproductive health of animals and humans with fertility issues stemming from GnRH system deficiency.

## Materials and methods

### Transgenic animals

dnFGFR mice were previously generated by Tsai et al. by targeting the expression of a *dnFGFR* transgene to GnRH neurons using a rat GnRH promoter (14, 16). This transgene encodes a truncated FGFR1 lacking an intracellular tyrosine kinase domain, and when bound to an FGF ligand, forms a nonfunctional heterodimer with endogenous FGFRs 1, 2, and 3, leading to attenuated FGF signaling in GnRH neurons (12, 14, 16). All dnFGFR breeding pairs were validated to be homozygous for the *dnFGFR* transgene by real-time quantitative PCR (qPCR) of genomic DNA using the 2^-ΔΔCT^ method (17, 18). Control mice with matching genetic backgrounds were generated by mating heterozygous dnFGFR siblings. Animals were housed in the University of Colorado Conventional Facility under a 12L:12D photoperiod and provided food and water *ad libitum*. All animal procedures were approved by the Institutional Animal Care and Use Committee at the University of Colorado Boulder.

### Housing conditions

Experimental male mice (control or dnFGFR) were weaned on postnatal day (PND) 20 and immediately paired with a same-sex (SS) male or opposite-sex (OS) female partner. Cage mates were matched for age (PND20-27) and relative body size. OS-housed animals were allowed to breed freely and raise pups (1-5 liters per cage) to closely mimic conditions studied previously (12). Pups generated in OS-housed cages were weaned and removed from the cage at PND20. Experimental animals and their cage mates were then sacrificed on PND320, after 300 days of cohabitation.

### Tissue collection

Mice were weighed, anesthetized with isoflurane vapor, and rapidly decapitated. Trunk blood was collected, left to coagulate on ice for 30 minutes, and centrifuged to generate serum samples. To isolate POA samples, whole brains were blocked using a prechilled mouse brain matrix to generate a brain slice spanning from 2 mm rostral to the optic chiasm (OC). The resulting slice was further trimmed with a transverse cut inferior to the medial septum, and two parasagittal cuts lateral to the internal capsule, generating a final POA sample. The hypothalamic samples were generated from the remaining tissue by a coronal cut at the rostral border of the mamillary body, two parasagittal cuts along the lateral borders of the hypothalamus, and a dorsal cut below the optic chiasm. The pituitary gland was also collected. The serum, POA, hypothalamus, and pituitary samples were flash frozen on dry ice and stored at -70°C for RNA isolation (POA) or quantification of GnRH peptide (hypothalamus) and gonadotropins (serum and pituitary) by radioimmunoassays (RIAs). Paired testes and seminal vesicles (SV) were removed and weighed at the time of sacrifice. Gonadosomatic index (GSI) and seminal vesicle somatic index (SVSI) were calculated by dividing the wet mass of both paired organs by the terminal body mass.

### Gonadotropin and GnRH radioimmunoassays

Luteinizing hormone (LH) and follicle-stimulating hormone (FSH) in the pituitary and serum samples were measured using specific RIAs as previously described (19). GnRH peptide in the hypothalamic extract was quantified using a previously validated GnRH RIA (19).

### RNA isolation and quantitative PCR for GnRH

Total RNA was isolated from the POA samples using the RNeasy Mini kit (Qiagen) according to the manufacturer’s instructions. The quality of total RNA was initially assessed by 260nm/280nm absorbance ratio and agarose gel electrophoresis. Synthesis of cDNA from 1μg of RNA was performed using the Superscript III first strand synthesis system (Invitrogen), and qPCR was performed using a SYBR green master mix (Roche Diagnostics). Detailed protocols for qPCR were previously described (12, 20). Briefly, samples were run in duplicates for *GnRH* or a housekeeping gene *hypoxanthine phosphoribosyltransferase* (*HPRT*) using primers in **Table 1**. Relative expression was quantified using the 2^-ΔΔCT^ method (17, 18).

**Table 1:** Primers for GnRH qPCR.

Target Gene	Forward Primer Sequence (5’-3’)	Reverse Primer Sequence (5’-3’)
*GnRH*	TCA GGG ATC TGC GAG GAG	GGG CCA GTG CAT CTA CAT
*HPRT*	AGC AGT ACA GCC CCA AAA TGG	TGC GCT CAT CTT AGG CTT TGT

### RNA-Seq

Total POA RNA was subject to additional quality control by the Qubit RNA Assay and the Agilent^®^ 2100 BioAnalyzer at Novogene (Sacramento, CA). Library preparation and next generation Illumina sequencing were then performed by Novogene as described (21). Libraries were sequenced on an Illumina Hiseq 2000 platform, and ~40 million 150 bp paired-end reads were generated per sample. Results of sequencing were received in the form of .bam files representing raw reads preprocessed and aligned (HISAT2) (22) to the mouse genome (NCBI GRCm38) (23) by Novogene using their in-house bioinformatics pipeline. Counts were generated from aligned reads using Rsubread (v2.0.1) (24) for R, and annotated with the NCBI refSeq annotation (25). Sample outlier detection and removal were performed using robust principal component analysis (PCA) on all raw gene counts using RRcov (v1.7-3) (26) for R. DESeq2 (v1.36.0) (27) for R was used as a means to generate normalized counts, perform differential expression analysis, and prepare a ranked order gene list for gene set enrichment analysis (GSEA). Differential expression analysis was initially performed with all experimental groups included in a single DESeq object. Resulting *p-*value histograms indicated a need for denoising by group separation (28), so each genotype (control and dnFGFR) was analyzed separately due to previously demonstrated differences in their response to OS housing (12). Genes were considered significant with adjusted *p* < 0.1 (29), and all scripts used were uploaded to a GitHub repository (https://doi.org/10.5281/zenodo.14511480). Data can be found in NIH GEO at Accession# GSE285006.

### GSEA

A ranked order list of genes was generated for GSEA (30, 31) using the results from differential expression analysis by multiplying the sign of the log fold change (LFC) of each gene by the negative log of its *p*-value. The ranked list was run in GSEA 4.3.2 against the Molecular Signatures Database (32) for biological processes (BPs), and individual results were considered significant at a false discovery rate (FDR) < 0.05. Significant GSEA results were presented as heatmaps using the ComplexHeatmaps (v2.16.0) package for R. A z-score (standard score) table was generated by comparing normalized counts to the population mean, and core enriched genes in each significant biological process were plotted.

### Statistical analysis of physiological parameters

Hypothalamic GnRH, pituitary gonadotropins, SVSI, and GSI were analyzed for the effects of genotype, housing condition, and genotype x housing interaction using 2-way ANOVA, followed by Šidák’s correction for multiple comparisons. Statistical significance was defined as *P* < 0.05, and all data were presented as mean ± standard error of the mean (SEM).

## Results

### Seminal vesicle somatic index and gonadosomatic index

**Figure 1:**
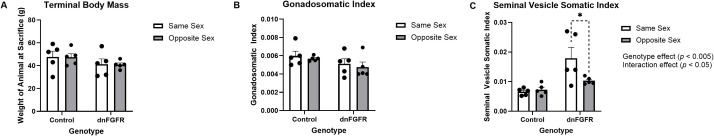
Terminal body mass **(A)**, gonadosomatic index (GSI) **(B)**, and seminal vesicle somatic index (SVSI) **(C)** of SS- and OS-housed control and dnFGFR mice at PND320. GSI and SVSI were determined by dividing the mass of the gonad or seminal vesicle, respectively, by the terminal body mass. Each bar represents mean ± SEM. Significant genotype and interaction effects were detected by two-way ANOVA in **(C)**. The bracket in dashed line represents significant difference detected by Šidák’s *post-hoc* test. *P < 0.05; *n* = 5/group.

SVSI and GSI represent the mass of paired seminal vesicles and testes divided by the terminal body mass, respectively, and are surrogate measures of circulating androgens and gonadotropins. Two-way ANOVA showed no significant effect of housing, genotype, or housing x genotype interaction on terminal body mass (**Figure 1A**) or GSI (**Figure 1B**). For SVSI, two-way ANOVA showed significant effects of genotype [*F*_(1, 16)_ = 14.25, *p* = 0.0017] and housing x genotype interaction [*F*_(1, 16)_ = 4.793, *p* = 0.0437], but not housing alone (**Figure 1C**). Two of 5 animals in dnFGFR SS group each had a swollen, fluid-filled SV that contributed to increased SVSI in this group (**Figure 1C**). Šidák’s *post-hoc* test also identified a significant difference between the SVSI of SS- and OS-housed dnFGFR animals (*p* = 0.024; **Figure 1C**).

### Gonadotropin radioimmunoassays

**Figure 2:**
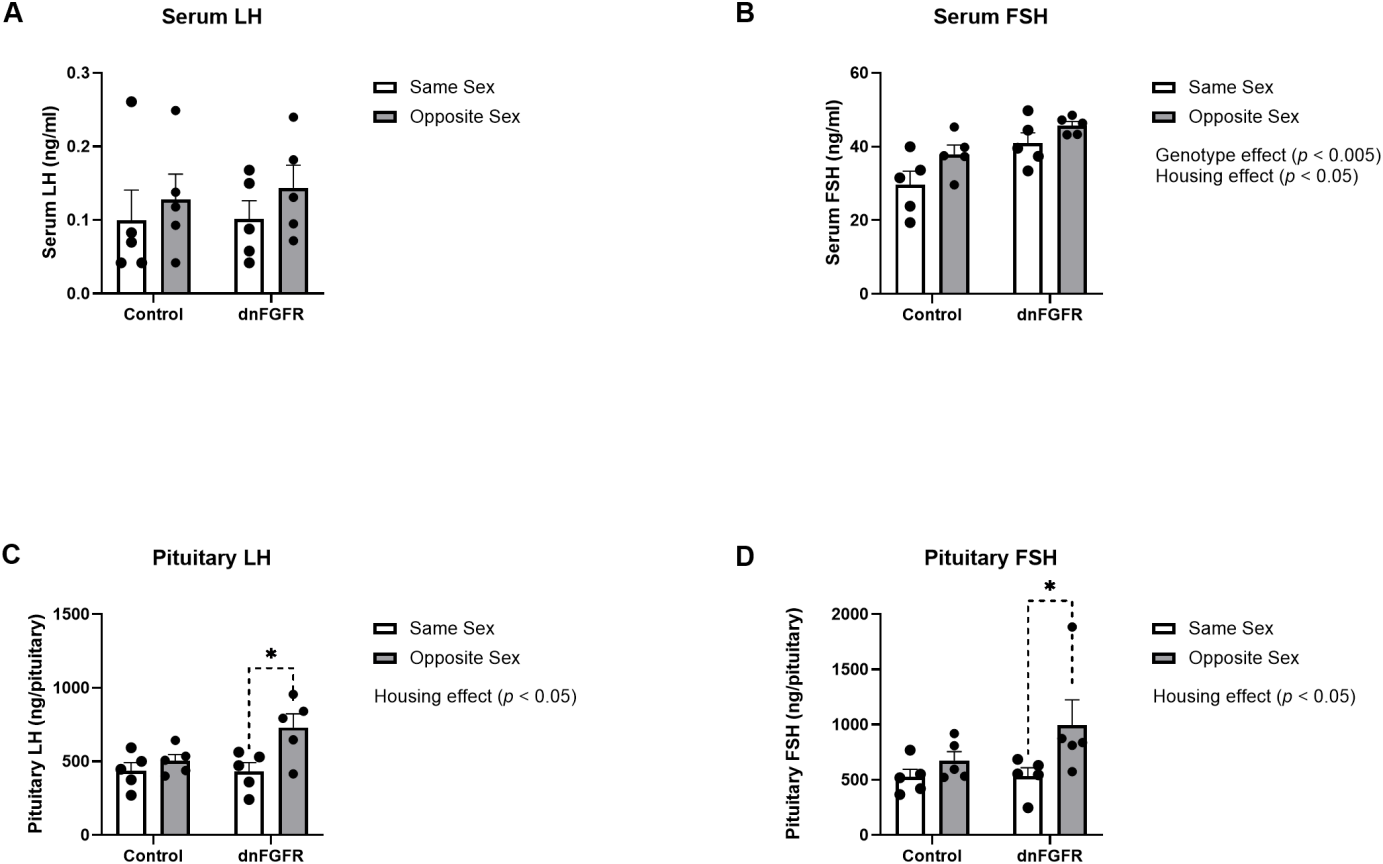
Serum LH **(A)**, serum FSH **(B)**, pituitary LH **(C)**, and pituitary FSH **(D)** in SS- and OS-housed control and dnFGFR mice at PND320. Each bar represents mean ± SEM. Significant genotype and/or housing effects were detected by two-way ANOVA in **(B–D)**. Brackets in dashed line represent significant differences detected by Šidák’s *post-hoc* test. *P < 0.05; *n* = 5/group.

Serum and pituitary LH and FSH were measured by specific RIAs. For serum LH, two-way ANOVA showed no significant effects of housing, genotype, or housing x genotype interaction (**Figure 2A**). For serum FSH, two-way ANOVA showed significant effects of housing [*F*_(1, 16)_ = 5.933, *p* = 0.0269] and genotype [*F*_(1, 16)_ = 12.62, *p* = 0.0027], but not housing x genotype interactions. However, Šidák’s *post-hoc* test did not identify significant differences among groups for serum FSH (**Figure 2B**). For pituitary LH, two-way ANOVA showed significant effects of housing [*F*_(1, 16)_ = 7.872, *p* = 0.0127], but not genotype or housing x genotype interaction. Šidák’s *post-hoc* test also identified a significant difference between the pituitary LH content of SS- and OS-housed dnFGFR animals (*p* = 0.0104). For pituitary FSH, two-way ANOVA showed significant effects of housing [*F*_(1, 16)_ = 5.465, *p* = 0.0327], but not genotype or housing x genotype interaction (**Figure 2C**). Šidák’s *post-hoc* test also identified a significant difference between the pituitary FSH content of SS- and OS-housed dnFGFR animals (*p* = 0.047; **Figure 2D**).

### Hypothalamic GnRH peptide content and GnRH mRNA expression

**Figure 3:**
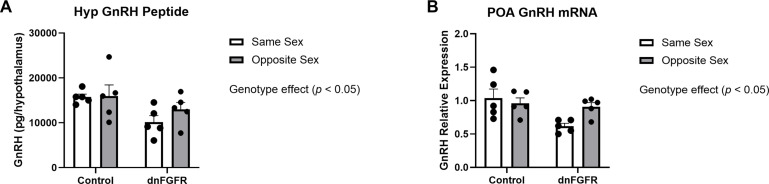
Hypothalamic GnRH peptide **(A)** and preoptic area *GnRH* mRNA **(B)** in SS- and OS-housed control and dnFGFR mice at PND320. Each bar represents mean ± SEM. Significant genotype effects were detected by two-way ANOVA in **(A, B)**. No significant differences were detected by Šidák’s *post-hoc* test. *n* = 5/group.

Two-way ANOVA showed an effect of genotype, but not housing or housing x genotype interaction, on both hypothalamic GnRH peptide [*F*_(1, 16)_ = 6.594, *p* = 0.0206] and *GnRH* expression in the POA [*F*_(1, 16)_ = 7.230, *p* = 0.0161] (**Figure 3**). Šidák’s *post-hoc* test did not identify specific differences among groups.

### Differential expression analysis

Control and dnFGFR animals were analyzed for differentially expressed genes (DEGs) in the POA of OS- versus SS-housed mice. In control mice, no DEGs were detected between SS- and OS-housed animals. In dnFGFR mice, OS housing resulted in 72 DEGs (22 upregulated and 50 downregulated) compared to SS housing (adjusted *p* < 0.1; **Supplementary Table 1**). Detailed per-mouse results are provided in **Supplementary Table 2**.

### Gene set enrichment analysis

Since only dnFGFR animals responded to OS housing through altered gene expression, we used GSEA (30, 31) to identify biological processes altered by housing conditions in the POA of dnFGFR mice. The results of differential expression analysis between SS- vs OS-housed dnFGFR mice were used to generate a ranked order list. This list was run against the biological processes from Molecular Signatures Database (32), and 20 gene sets (16 positive and 4 negative) were found to be significantly enriched (FDR *q*-value < 0.05; **Table 2**). Of these, 5 positively enriched and 4 negatively enriched sets showed a Family-wise error rate (FWER) of *p*-value < 0.05 (**Table 2**).

**Table 2:** Biological processes altered by opposite-sex housing.

Upregulated Biological Processes				
Biological Process	Size	Enrichment Score	FDR q-val	FWER p-val
CYTOPLASMIC TRANSLATION	142	0.5695814	0.003133532	0.003
URONIC ACID METABOLIC PROCESS	20	0.7947593	0.007231531	0.014
CELLULAR GLUCURONIDATION	15	0.85748434	0.008559899	0.025
PROTEIN FOLDING	166	0.5359303	0.006679071	0.026
ESTROGEN METABOLIC PROCESS	32	0.6828365	0.008231449	0.04
DE NOVO PROTEIN FOLDING	37	0.6713271	0.011341961	0.066
PROTEIN LOCALIZATION TO ENDOPLASMIC RETICULUM	73	0.58853483	0.011782042	0.08
CHAPERONE MEDIATED PROTEIN FOLDING	63	0.59172297	0.01420114	0.109
PEPTIDE ANTIGEN ASSEMBLY WITH MHC PROTEIN COMPLEX	15	0.8083554	0.016068306	0.138
SPHINGOID METABOLIC PROCESS	22	0.7366126	0.015182643	0.143
PROTEIN K11 LINKED UBIQUITINATION	29	0.67458177	0.020018505	0.201
ESTABLISHMENT OF PROTEIN LOCALIZATION TO ENDOPLASMIC RETICULUM	46	0.62805116	0.02076389	0.221
ANTIGEN PROCESSING AND PRESENTATION OF PEPTIDE OR POLYSACCHARIDE ANTIGEN VIA MHC CLASS II	26	0.6932599	0.022364037	0.251
ENDOPLASMIC RETICULUM TO GOLGI VESICLE MEDIATED TRANSPORT	117	0.5154504	0.02980593	0.337
COTRANSLATIONAL PROTEIN TARGETING TO MEMBRANE	19	0.7341076	0.03885864	0.428
RIBOSOMAL LARGE SUBUNIT BIOGENESIS	67	0.55397636	0.043474242	0.486

Downregulated Biological Processes				
Biological Process	Size	Enrichment Score	FDR q-val	FWER p-val
CHEMICAL SYNAPTIC TRANSMISSION POSTSYNAPTIC	103	-0.5904774	0.014679906	0.015
POSITIVE REGULATION OF EXCITATORY POSTSYNAPTIC POTENTIAL	42	-0.6857255	0.007339953	0.015
NEUROMUSCULAR PROCESS CONTROLLING BALANCE	70	-0.61849564	0.01170511	0.036
NEUROMUSCULAR PROCESS	191	-0.5421305	0.009513867	0.039

**Figure 4:**
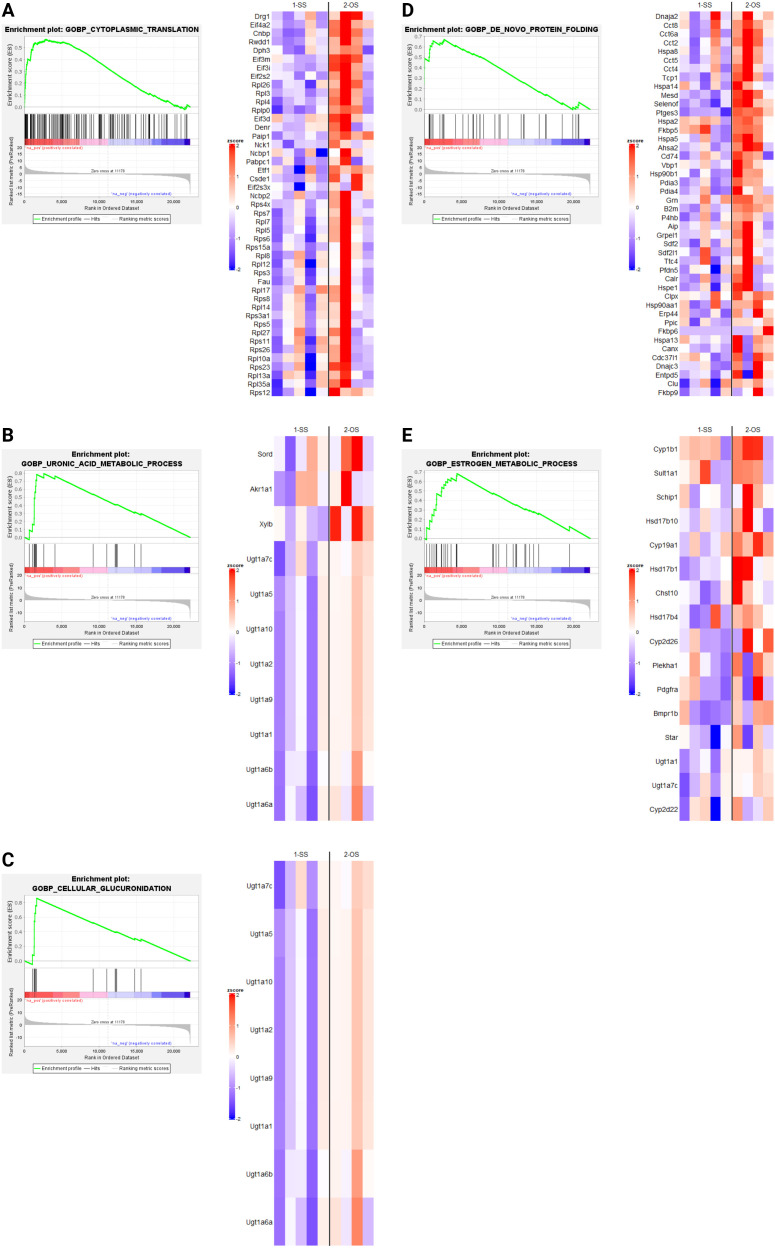
Upregulated biological processes (BPs) revealed by GSEA in SS- and OS-housed dnFGFR mice. Each panel **(A–E)** represents a biological process found to be significantly positively enriched by GSEA (FWER < 0.05) followed by a heatmap of the core enriched genes of each BP, identified by GSEA leading edge analysis. *n* = 4-5/group after sample outlier detection. Created in BioRender. Akonom, T. (2024) https://BioRender.com/o85p895.

**Figure 5:**
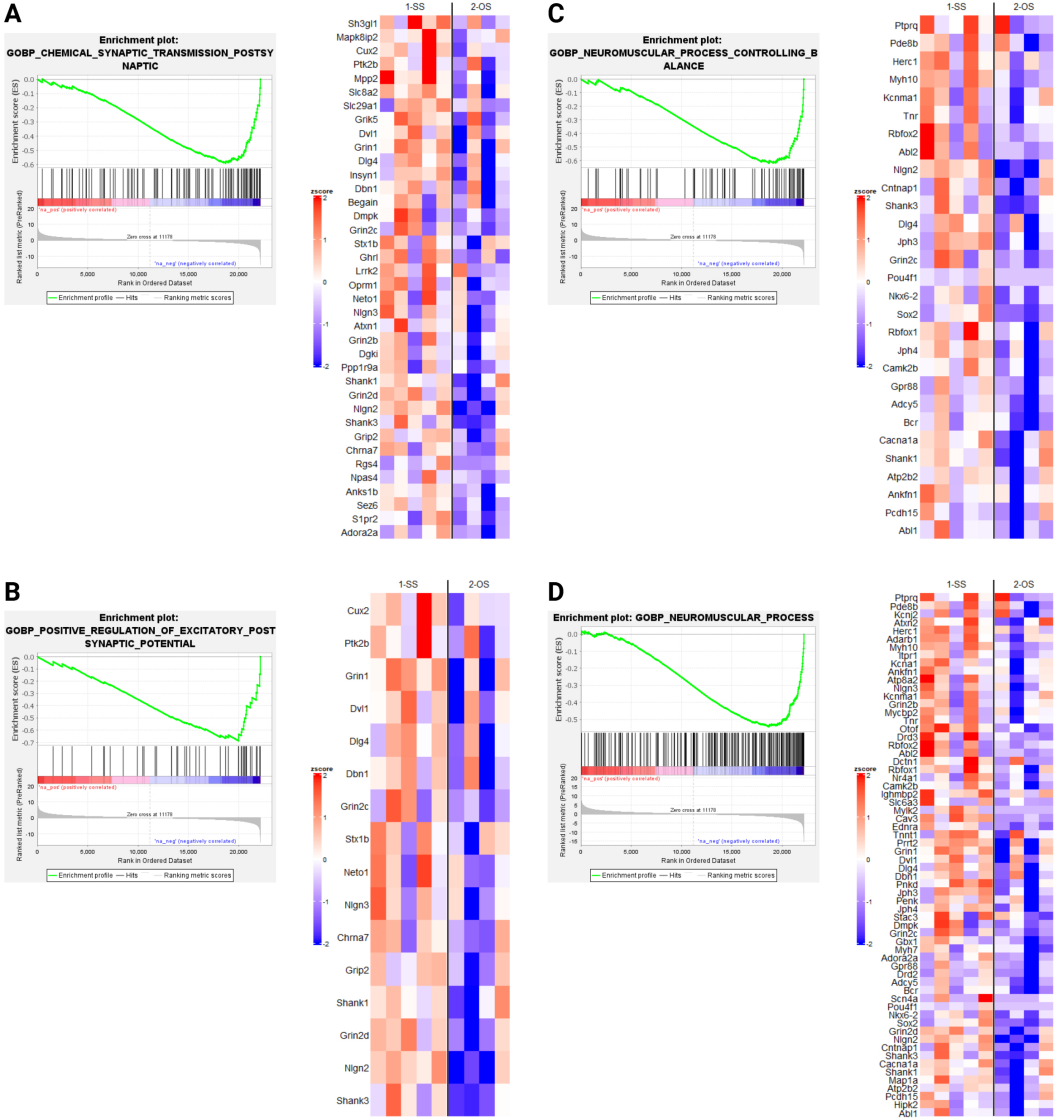
Downregulated biological processes (BPs) revealed by GSEA in SS- and OS-housed dnFGFR mice. Each panel **(A–D)** represents a biological process found to be significantly negatively enriched by GSEA (FWER < 0.05) followed by a heatmap of the core enriched genes of each BP, identified by GSEA leading edge analysis. *n* = 4-5/group after sample outlier detection. Created in BioRender. Akonom, T. (2024) https://BioRender.com/e36u652.

GSEA revealed several notable biological processes altered by OS housing in dnFGFR mice. Processes positively enriched (**Figure 4**) included those involved in protein translation and folding (**Figures 4A, D**). In addition, three processes associated with steroid metabolism via glucuronic acid conjugation (**Figures 4B, C, E**) were positively enriched. Lastly, core enriched genes also included multiple members of the cytochrome P450 superfamily involved in the synthesis and breakdown of estrogens (**Figure 4E**). All negatively enriched biological processes were found to represent excitatory neural processes (**Figure 5**). These results suggest an effect of OS housing on the *de novo* synthesis and metabolism of neurosteroids locally within the POA as well as an overall decrease of excitatory neurotransmission in this region.

## Discussion

The current study revealed several prominent effects of OS housing on reproductive parameters and the POA transcriptional landscape. First, OS housing significantly altered pituitary and serum gonadotropin profiles and mitigated SV fluid accumulation, a marker of reproductive senescence, in dnFGFR mice (33). Second, OS housing of dnFGFR mice altered the expression of several biological processes related to steroid metabolism and excitatory neural processes. These results revealed biological pathways in the POA activated concomitantly with the restoration of the HPG axis in dnFGFR mice, providing a glimpse into potential mechanisms underlying the plasticity of the GnRH system.

dnFGFR mice were previously reported to harbor an age-dependent decline of detectable GnRH-ir neurons, leading to multiple reproductive defects, including delayed puberty, earlier reproductive senescence, and decreased fertility (14). The cause for this neuronal decline was likely neuronal de-differentiation instead of neuronal death, leading to the loss of *GnRH* expression (34). Although the decline in detectable GnRH-ir neurons and several other reproductive defects could be ameliorated by long periods of cohabitation with an OS partner (12), mechanisms underlying these OS-associated benefits were unclear. Of note, our current data showed that changes in DEGs as well as anatomical and endocrine parameters occurred only in OS-housed dnFGFR mice but not control mice. This was consistent with previous observations that the GnRH system in control animals was unaffected by OS housing (12). One parsimonious explanation is a ceiling effect in which the reproductive brain in control animals was already optimal and could not be functionally enhanced further.

Anatomical indicators measured in this study included terminal body mass, GSI, and SVSI. OS housing had no significant effect on terminal body mass or GSI in either genotype. However, the SVSI of SS-housed dnFGFR males was significantly larger than OS-housed dnFGFR males. This result was partly driven by two SS-housed dnFGFR mice each with a fluid-filled SV, a common pathology in senescent male mice (33). Although SV enlargement did not occur in every SS-housed dnFGFR animal, the complete absence of this pathology in OS-housed dnFGFR mice and control mice suggested dnFGFR males were prone to accelerated reproductive aging, and OS housing significantly delayed this aging.

Our data showed OS housing impacted serum and pituitary gonadotropins differently between genotypes. For serum LH, there were no discernible differences between genotypes and housing conditions, likely due to the dynamic nature of LH pulsatility that could not be captured by a single measurement (35). Serum FSH was significantly elevated in dnFGFR mice compared to controls, presumably reflecting reduced negative feedback from decreased testicular steroidogenesis as previously reported in dnFGFR males (12). Two-way ANOVA also detected a significant effect of housing on serum FSH, but *post-hoc* tests failed to identify differences between specific groups. On the contrary, results from pituitary gonadotropins unambiguously demonstrated a stimulatory effect of OS housing on dnFGFR mice. Lastly, two-way ANOVA detected significant decreases in GnRH peptide and expression in dnFGFR animals compared to controls, consistent with previous reports on the decline of GnRH-ir neurons in dnFGFR mice (12, 14). Although two-way ANOVA failed to detect a significant effect of housing on GnRH peptide and transcript, OS-housed dnFGFR mice showed a trend towards increased *GnRH* expression compared to SS-housed counterparts (**Figure 3B**), suggesting enhanced GnRH neuronal activation in progress. In summary, anatomical and endocrine measurements confirmed that, at the time of sacrifice, dnFGFR mice had already initiated a myriad of beneficial changes within the HPG axis in response to OS housing. Although these changes were somewhat incomplete, they signified a GnRH system in transition toward functional restoration. Importantly, they supported transcriptional changes detected in the POA (see below) as contributors to the functional recovery of the GnRH system.

Our DEG analysis revealed OS housing had no effect on gene expression in control mice. Previous studies in wildtype mice have reported benefits of OS housing on reproduction of older males (10) but not younger males. We posit that wild-type males may not respond to beneficial cues of OS housing until older, past PND320, due to their relatively stable lifetime GnRH-ir neuronal population (36) and healthy reproductive capacity within the first year. On the contrary, OS housing induced 77 DEGs in dnFGFR males, which exhibited a significant decline in GnRH-ir neuronal as early as PND30 (14). Detectable transcriptional changes in dnFGFR males but not control animals suggests that GnRH plasticity is more pronounced in animals with a deficient GnRH system, even when exposed to the same environmental modulation. This observation is consistent with observations in humans, where partial reversibility of hypogonadotropic hypogonadism has been reported under specific conditions (37). At first glance, 77 DEGs appeared relatively few considering the many sensory inputs that may impact the POA (2). A less likely explanation for this relatively low number of DEGs is that OS housing had only a modest impact on transcriptional changes. However, a more likely explanation is the diversity of cell types within the POA (38) has diluted gene expression changes in our bulk RNA-Seq analysis. Most afferent projections to the POA convey different modalities of somatosensory information during proceptive and consummatory phases of sexual behavior (2, 39). While the POA is where these diverse stimuli are functionally integrated, each pathway impacts reproduction and reproductive behaviors in different ways (2). For example, the three dopaminergic pathways regulating sexual behavior (nigrostriatal, mesolimbic, and periventricular/zona incerta) all provide input to the POA but are involved in different stages of reproductive behavior (2). These complex neurocircuits likely triggered the change of many genes in OS-housed mice. In bulk RNA-Seq analysis, collecting less robust changes across many genes could increase the threshold for a gene to be identified as a DEG after correcting for multiple comparisons, thereby decreasing detectable DEGs in response to OS housing. As such, a more meaningful interpretation of our DEG analysis required a follow-up analysis with GSEA which is agnostic to multiple testing corrections.

GSEA indicated OS housing in dnFGFR mice resulted in both upregulated (**Figure 4**) and downregulated (**Figure 5**) biological processes (BPs). Upregulated BPs included “cytoplasmic translation” (**Figure 4A**) and “*de novo* protein folding” (**Figure 4D**), suggesting an uptick in POA activity through increased production and assembly of functional proteins. This observation is consistent with previous findings that local protein synthesis in the POA was enhanced through hormonal and environmental stimulus (40), but is seemingly at odds with two downregulated BPs representing “chemical synaptic transmission postsynaptic” (**Figure 5A**) and “positive regulation of excitatory postsynaptic potential” (**Figure 5B**). That said, the POA is home to ~70 different neuronal populations with 43 inhibitory, 23 stimulatory, and ~4 hybrid cell types (38). In this sense, inhibitory neurons dominate POA neurotransmission. It stands to reason that downregulation of neuronal excitability in the POA could signify an overall activation of inhibitory POA neurons to suppress inhibitory POA interneurons and/or disinhibit downstream targets controlling sexual behavior and the reward circuit (2). The overall consequence may be the activation of downstream POA targets to reap the additional benefits of OS.

The remaining upregulated BPs (**Figures 4B, C, E**) were related to steroid hormone metabolism, including those involved in glucuronic acid conjugation. Sex steroids are the principal hormones driving reproductive behavior and the accumulation of sexual experience (2). The accumulation of sexual experience leads to a progressive maturation of the POA through dendritic spine maturation and is associated with an increase in sexual motivation as well as beneficial reproductive behaviors (2, 41). For example, sexually experienced rats require less mounts and intromissions, as well as less time to reach ejaculation, leading to better reproductive outcomes (42). In mice, these effects required the presence of 17β-estradiol (E2), dihydrotestosterone, or testosterone (43, 44). Astrocyte- and neuron-derived E2 has been shown to have a critical role in neuroprotective and neuroplastic responses to ischemic brain injury (45), as well as regulating synaptic plasticity and cognitive functions in both sexes (46). Interestingly, *CYP19A1*, responsible for the conversion of testosterone to estradiol (45), is an upregulated gene in the BP “estrogen metabolic process”. This observation suggested the possible involvement of brain-derived E2 in the restoration of GnRH-ir neurons. GnRH neurons do not express estrogen receptors (47), but stimulatory afferents of GnRH neurons in the POA region, such as the rostral kisspeptin neuronal population, are highly sensitive to estrogen signaling (47). Further, estrogens within the POA/hypothalamus were reported to upregulate insulin-like growth factor 1 (IGF-1) signaling that is stimulatory to GnRH synthesis and release (48–50). Lastly, there is an upregulation of GnRH production in dnFGFR males during puberty when gonadal steroids are increased (51), suggesting a robust plasticity for this system to compensate for its deficiency. These data led us to hypothesize that the local activation of estrogen signaling may indirectly rescue the defective GnRH-ir neurons through stimulatory afferents such as kisspeptin neurons. We posit that these BPs related to steroid metabolism could alter the turnover of POA-derived steroids, thereby enhancing local neuroprotective and neuroplastic effects on GnRH neurons in OS-housed mice.

An important observation is that DEGs and changes in BPs were not consistently associated with OS housing since OS-housed controls did not exhibit these changes. Instead, they were more closely associated with endocrine and anatomical changes of reproductive recovery seen in dnFGFR mice. In other words, reproductive improvement was unlikely due to the simple exposure of a male to a female. Rather, it resulted more directly from long-term changes in gene expression networks detected here and from interactions among the organism’s genotype, phenotype and environment.

To summarize, this study demonstrated transcriptional changes in the POA induced by OS housing in an FGF signaling-deficient mouse. These changes coincided with ongoing improvements in the HPG axis of dnFGFR mice, suggesting a causal relationship between BPs detected by GSEA and the restoration of the defective GnRH system. Importantly, an increase in brain-derived estradiol synthesis and turnover occurred concomitantly with the restoration of the HPG axis in OS-housed dnFGFR mice, suggesting a possible role of local estrogen signaling in the neuroprotection of the GnRH system. With the implication of E2 as a key player in the plasticity of the GnRH system, a promising and novel direction for the improvement of this necessary and resilient system has been revealed.

## Data Availability

The datasets presented in this study can be found in online repositories. The names of the repository/repositories and accession number(s) can be found in the article/**Supplementary Material**.
